# Is it really descemetocele? Morphology of extremely thin membrane that remained after severe corneal melting: a case report

**DOI:** 10.1007/s00795-024-00405-z

**Published:** 2024-10-01

**Authors:** Yasser Helmy Mohamed, Masafumi Uematsu, Mao Kusano, Takashi Kitaoka, Teruo Nishida

**Affiliations:** 1 Department of Ophthalmology and Visual Sciences, Graduate School of Biomedical Sciences, Nagasaki University, 1-7-1 Sakamoto, Nagasaki, Nagasaki 852-8501 Japan; 2 Department of Ophthalmology, Graduate School of Medicine, Yamaguchi University, Ube, Yamaguchi Japan

**Keywords:** Descemetocele, Myofibroblast, Epithelial–mesenchymal transition (EMT), Transmission electron microscopy (TEM), Case report

## Abstract

The aim of this study was to report transmission electron microscopic findings of a case with whole corneal descemetocele following infective corneal ulcer for the first time in literature. A 72-year-old male patient presented with infective corneal ulcer. After resolution of the infection, corneoscleral transplantation was performed. The excised very thin corneal membrane was processed for transmission electron microscopic examination. Transmission electron microscopic examination of the specimen revealed many layered structures that consisted of two different types of cells. The first type consisted of lighter staining polygonal cells, while the second consisted of elongated cells with relatively dense staining. All cells were connected with a large number of gap or adherens junctions with intercalation of the cell membranes of adjacent cells. A haphazard distribution of cytoplasmic microfilaments were also observed in all of the cell types. There was no evidence of the presence of endothelial cells throughout the specimen. There was also no evidence of Descemet membrane presence except for a small part adjacent to iris tissue that contained some melanosomes. Although we clinically diagnosed descemetocele, Descemet membrane was not present at the electron microscopic level, and thus, the expression “descemetocele” is inappropriate.

## Background

Descemetocele involves herniation or anterior bulging of an intact Descemet membrane (DM) through a defect of the overlying corneal stromal and epithelial layers [1]. This rare, but serious complication of corneal ulceration is characterized by thinning of the stroma to such an extent that the DM is the only layer maintaining the integrity of the globe [2, 3]. Due to the lack of adequate tensile strength, the DM herniates anteriorly and appears like a cyst protruding through the overlying corneal stromal defect, hence the use of the term, descemetocele, to refer to this clinical condition [1]. Various ocular and systemic abnormalities can lead to the formation of descemetocele, including microbial keratitis, neurotrophic keratopathy, dry eye disorders, and corneal inflammation associated with immune-mediated disorders [4]. Infectious corneal ulcer can especially lead to significant melting of the corneal stromal tissue and must be treated with prompt intervention to restore the ocular structural integrity [5].

Previously, we have reported a rare clinical case of severe infectious corneal melting that left a very thin transparent membrane over the entire corneal area. We suspected this membrane to be DM and diagnosed it as “whole corneal descemetocele” [6]. However, we could not use light microscopy or immunohistochemistry to examine the specimen due to its small size. Therefore, we tried to use transmission electron microscopy (TEM) to examine the tiny specimen to clarify the morphology of the descemetocele in this case. To the best of our knowledge, there is no literature that has described TEM examination or any light microscopic study of a corneal descemetocele. In the present study, we report on the pathological findings conducted at the electron microscopic level of the membrane, which we clinically diagnosed as descemetocele.

## Case presentation

**Fig. 1:**
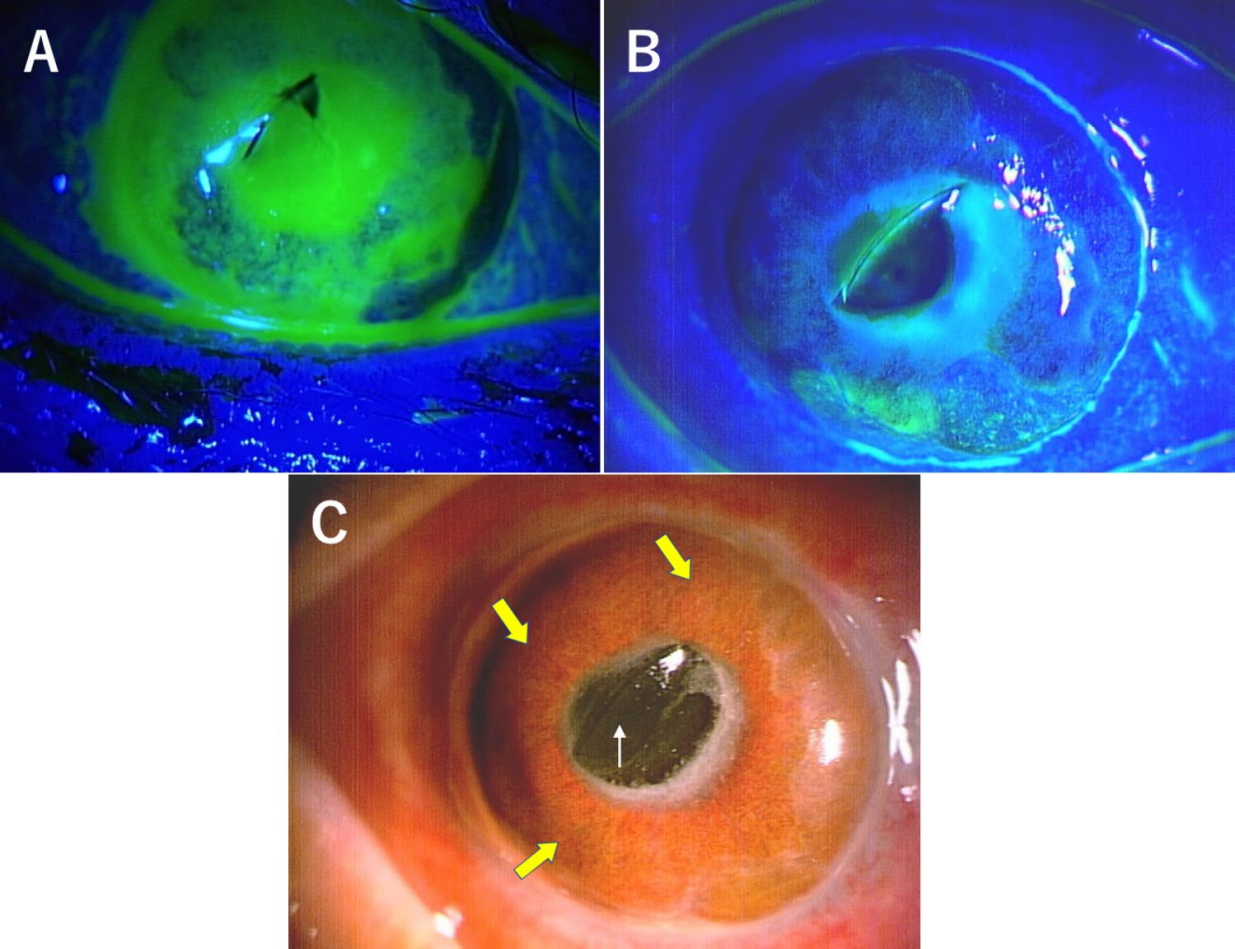
**A** Right corneal ulcer stained greenish with fluorescein involving nearly the whole cornea which was almost completely melted down with remaining Descemet’s membrane. **B** Right cornea showed resolution of ulceration and disappearance of greenish staining with fluorescein except for a few scattered spots. **C** Right corneal descemetocele (yellow arrows) protruding forward, and the intraocular lens (white arrow) partially protruding from the pupil and indenting the Descemet’s membrane

In our previously published clinical case report of whole corneal descemetocele after infective corneal ulcer, we described an old male farmer presented with right infective corneal ulcer (positive fluorescein staining) involving nearly the entire cornea (Fig. 1A), which was almost completely melted down with the remaining Descemet’s membrane (DM) [6]. After the corneal inflammation had settled down within 2 weeks of hospitalization and intensive treatment, the cornea showed negative staining for fluorescein (Fig. 1B) observed over the transparent and thin remaining membrane which was indented by protruding intraocular lens (Fig. 1C). Thus, we performed corneoscleral transplantation plus excision/transplantation of the corneal limbus. This study was approved by the Institutional Review Board of Nagasaki University Hospital (approval number 23082143) and the patient involved in the study provided informed consent.

### Transmission electron microscopy (TEM)

**Fig. 2:**
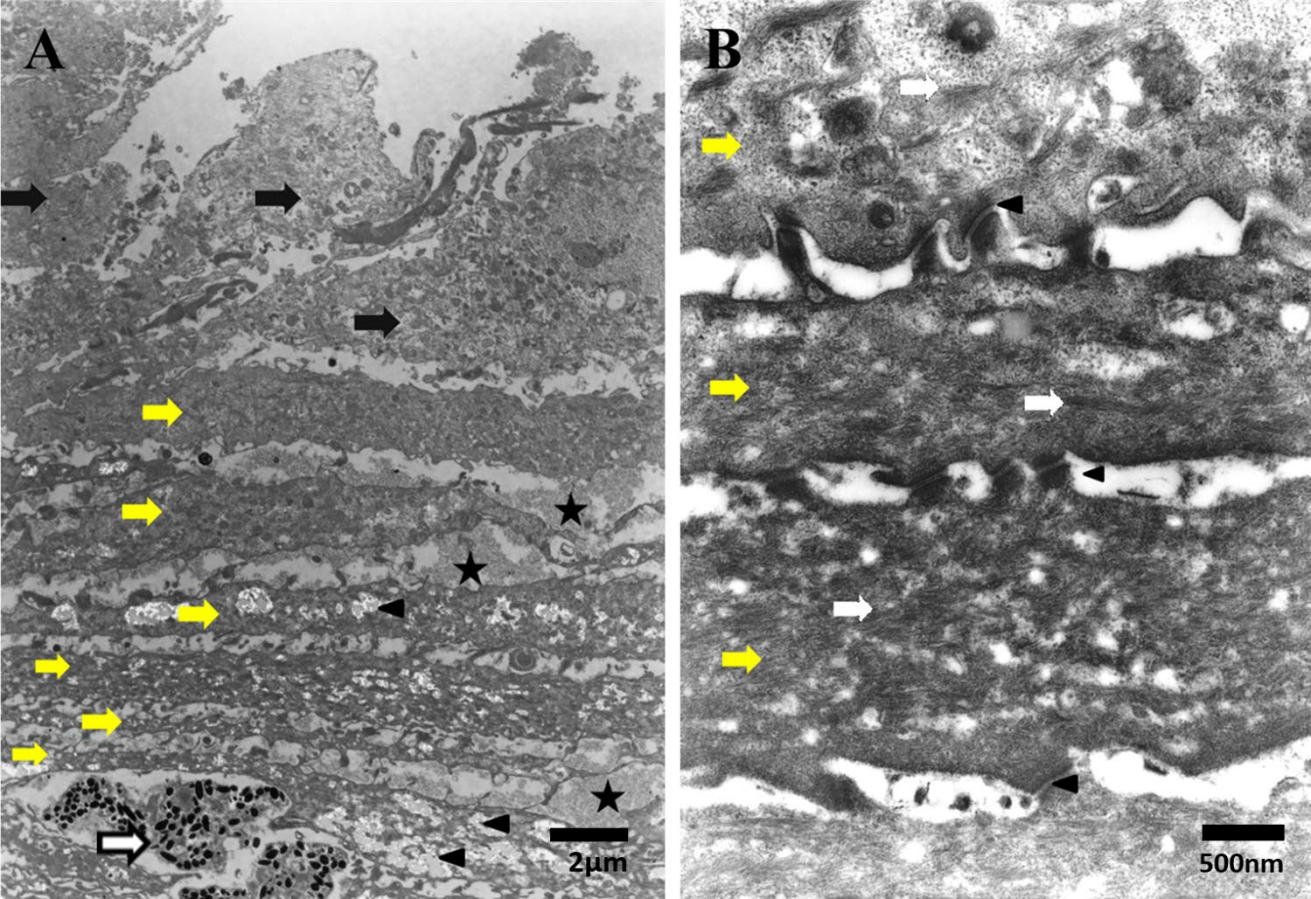
**A** TEM image showing the many layered structures that consist of two different types of cells. The first type of cell consists of lighter staining polygonal cells (black arrows), while the second is formed of elongated cells (yellow arrows) with relatively dense staining. (Star = ECM; arrowhead = intracellular spaces and white arrow = macrophage) (Bar = 2 µm). **B** TEM image showing the parallel layers of elongated cells (yellow arrows) attached to each other with multiple gap junctions (arrowheads). (White arrows = stress fibers) (Bar = 500 nm)

**Fig. 3:**
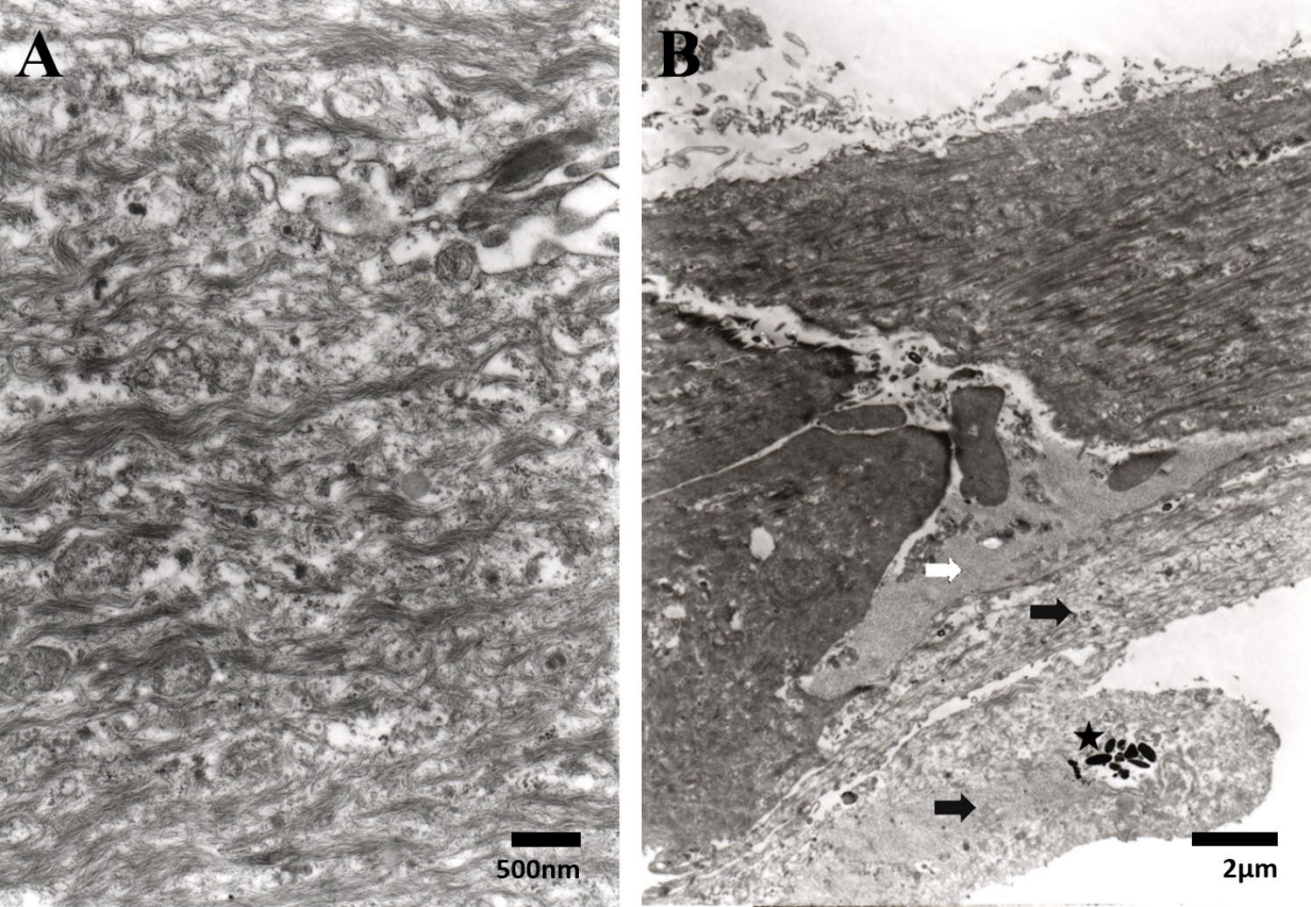
**A** TEM image showing the haphazard distribution of cytoplasmic microfilaments in one elongated cell (Bar = 500 nm). **B** TEM image showing part of the remanent of Descemet’s membrane (white arrow) that is attached to atrophied featureless iris tissue (black arrows), which contains melanosomes (star) (Bar = 2 µm)

The excised corneal descemetocele attached to the iris was fixed with 4% glutaraldehyde in 0.05 M cacodylate buffer for 1 h, washed in 0.05 M cacodylate buffer overnight, postfixed with osmium tetraoxide in veronal acetate buffer for 1 h, dehydrated in a series of ethanols, and embedded in Luveac 812. Ultrathin sections were cut with a Porter-Blum MT2 microtome, stained with uranyl acetate and lead citrate and examined with TEM (Hitachi H300, Hitachi, Ibaragi, Japan). TEM examination of the specimen revealed many layered structures that consisted of two different types of cells attached to each other (Fig. 2A). The first type of cells was the lighter staining polygonal cells, while the second type consisted of elongated cells with a relatively dense staining (Fig. 2A). Some polygonal cells had a disintegrated cell membrane with dispersed cellular debris. All cells were connected with a large number of gap or adherens junctions, with intercalation of the cell membranes with adjacent cells (Fig. 2B). Both types of cells contained a haphazard distribution of cytoplasmic microfilaments, especially the elongated cells (Fig. 3A). Elongated cells were arranged in parallel layers with interstitial deposition of ground substance (extracellular matrix, (ECM)), as well as with lighter dense ground substances. Between the elongated cells that contained large amounts of debris, macrophages were detected (Fig. 2A). There was no evidence of the presence of endothelial cells throughout the specimen. Also, there was no evidence of the presence of DM, with the exception for a small remanent part adjacent to the atrophied disorganized iris tissue that contained some melanosomes (Fig. 3B).

## Discussion and conclusions

To the best of our knowledge, this is the first electron microscopic study of a case of whole corneal descemetocele to be reported in the literature. Although this case was clinically diagnosed as “whole corneal descemetocele,” the main components of this membrane were composed of layers of cells filled with cytoplasmic microfilaments that were connected through gap and tight junctions. These cells, in conjunction with other components, may have formed a membrane that clinically resembles DM.

DM is an 8–10 µm thick, transparent, elastic, acellular membrane that is secreted by endothelial cells [3, 7]. It is relatively resistant to proteolysis and biomechanical stress and protects the endothelium from destructive stromal processes [8]. Dua et al. [9] have reported the existence of an acellular pre-Descemet layer (PDL) in the human cornea, which is composed of 5–8 lamellae of collagen fibrils. Based on the presence of this layer, Narang et al. [7] described a separate clinical entity known as predescemetocele.

While descemetocele is a herniation of the bare DM, in predescemetocele, progressive corneal ulceration spares the PDL in addition to DM. However, these authors based their findings on only anterior segment OCT and concluded that histopathology and electron microscopy were required to confirm this hypothesis. In another study that used ultrastructural and immunohistochemical analysis of the normal posterior corneal stroma, the authors concluded that there was no distinctive acellular pre-DM stromal zone that would justify using the term “layer” [10].

In either condition (presence or absence of a PDL), all the previous studies had no doubt regarding the presence of DM. In the present case, although the patient was clinically diagnosed as “whole corneal descemetocele,” TEM could not determine the presence of either DM or PDL throughout the specimen with the exception for a small remanent part of DM adjacent to the atrophied disorganized iris tissue. Furthermore, there was no evidence of the presence of the endothelial layer.

In the present case, from a clinical perspective, the patient had an infective corneal ulcer. Since the ulcer resolved after treatment and there was negative staining for fluorescein, this indicated there was epithelial regeneration. Thus, based on the TEM images, the polygonal cells are similar to epithelial cells, which are supposed to have migrated from limbal stem cells. Regarding the elongated cells, these were speculated to be fibroblasts and/or myofibroblasts that contained condensed cytoplasmic microfilaments.

The relatively elongated myofibroblasts (20–30 µm long) possess a highly developed cytoskeleton, including a specific network of actin isoforms and intermediate filaments. Furthermore, they are highly metabolically active, which allows them to maintain the homeostasis of the ECM during tissue repair processes [11]. This is due to its ability to contract wounds, secrete ECM, and generate adhesion structures with the surrounding substrate [12]. Myofibroblasts are fibroblastic cells that have ultrastructural and physiological characteristics of smooth muscle cells, such as prominent intracellular microfilament bundles (stress fibers) and contractile responses to smooth muscle agonists [13].

Until recently, it was believed that all myofibroblasts in the cornea originated from corneal precursor cells, namely keratocytes or corneal fibroblasts, based on the observation that corneal fibroblasts transform into myofibroblasts when treated with transforming growth factor (TGF) β in vitro [14, 15]. Bone marrow may also be a source of myofibroblasts via circulating stem cells and/or fibrocytes [16]. Lastly, it has also been suggested that the process of epithelial– or endothelial–mesenchymal transition (EMT), which results from a dedifferentiation of epithelial or endothelial cells, can in some situations be the origin of myofibroblasts [17].

EMT is a pivotal process that plays a key role in physiological and pathological events, such as embryogenesis, wound healing, and cancer development. The EMTs associated with wound healing, tissue regeneration, and organ fibrosis are classified as type-2 EMTs [18, 19].

Recent studies of corneal wound healing suggest that activated corneal keratocytes develop myofibroblast-like characteristics including a putative contractile apparatus comprised, in part, of intracellular microfilament bundles (stress fibers) containing F-actin, myosin, and α-actinin [20]. Furthermore, as shown by Petroll et al., stress fibers within corneal myofibroblasts undergo temporal changes in organization that support a muscle like, contractile wound healing mechanism [21]. Other studies have revealed that stress fibers generate tension across the corneal wound and lead to tissue organization that helps to enhance the healing process [12, 13, 22].

We believe and agree with the previous studies that the elongated cells are myofibroblasts, which contain intracellular alpha-smooth muscle actin (α-SMA) microfilaments, and exert strong contractile forces on the ECM, thereby enabling wound closure, restoring corneal integrity, and helping to resist perforation.

Hasty and Hay demonstrated that both gap junctions and tight junctions may be present between corneal keratocytes that form a syncytial network [23]. These findings have been confirmed by other studies that examined normal and trypsin-digested rabbit corneas by transmission and scanning electron microscopy [24, 25]. This network may serve as a communication system between keratocytes in different regions of the cornea, thereby allowing for a coordinated response to injury, infiltration, or infection.

Although the first phase of tissue repair is mainly devoted to inflammation, throughout the tissue repair process, inflammatory cells, and particularly macrophages, which secrete TGF-β1 and proteases, orchestrate the relationships between the myofibroblast and the ECM. In particular, this involves macrophages that are in close contact with contractile myofibroblasts that modulate both ECM composition and biophysical state [26, 27]. As was reported in other studies, the present results also demonstrated that macrophages were in close contact with myofibroblasts.

There were some limitations in the present study. First, there is a possibility that the DM stacked on the iris was not sufficiently removed during the surgery. However, the images we obtained showed the iris tissue was adjacent to the corneal membrane, which confirms that the specimen contained all layers of the cornea. In addition, the specimen itself was tiny and limited to evaluation by only TEM. Second, we do not know if the TEM findings are specific to a whole corneal thinning case or possibly related to every type of corneal thinning (localized or diffuse). Light microscopy and immunohistochemistry evaluations will additionally be required to further elucidate its pathology.

In summary, findings in the present case suggest that the infective keratitis and its proteolytic enzymes dissolved the corneal structure including the DM, endothelium, Bowman’s layer, epithelium, and major part of the stroma. The epithelium during the healing stage regenerated and subsequently covered most of the corneal ulcer. In addition, elongated cells with cytoplasmic microfilaments appear to form a membrane that clinically resembles the DM trying to restore corneal integrity and which resists perforation.

We concluded that our case clinically diagnosed as “whole corneal descemetocele” is not a real descemetocele confirmed with our electron microscopic study. Further studies and accumulation of similar cases will need to be conducted to confirm our findings.

## Data Availability

The datasets used during the current study are available from the corresponding author on reasonable request.
